# Early Metabolic Defects in Dexamethasone-Exposed and Undernourished Intrauterine Growth Restricted Rats

**DOI:** 10.1371/journal.pone.0050131

**Published:** 2012-11-16

**Authors:** Emmanuel Somm, Delphine M. Vauthay, Audrey Guérardel, Audrey Toulotte, Philippe Cettour-Rose, Philippe Klee, Paolo Meda, Michel L. Aubert, Petra S. Hüppi, Valérie M. Schwitzgebel

**Affiliations:** 1 Department of Paediatrics, University of Geneva School of Medicine, Geneva, Switzerland; 2 Department of Cell Physiology and Metabolism, University of Geneva School of Medicine, Geneva, Switzerland; Cincinnati Children's Hospital Medical Center, United States of America

## Abstract

Poor fetal growth, also known as intrauterine growth restriction (IUGR), is a worldwide health concern. IUGR is commonly associated with both an increased risk in perinatal mortality and a higher prevalence of developing chronic metabolic diseases later in life. Obesity, type 2 diabetes or metabolic syndrome could result from noxious “metabolic programming.” In order to better understand early alterations involved in metabolic programming, we modeled IUGR rat pups through either prenatal exposure to synthetic glucocorticoid (dams infused with dexamethasone 100 µg/kg/day, DEX) or prenatal undernutrition (dams feeding restricted to 30% of *ad libitum* intake, UN). Physiological (glucose and insulin tolerance), morphometric (automated tissue image analysis) and transcriptomic (quantitative PCR) approaches were combined during early life of these IUGR pups with a special focus on their endocrine pancreas and adipose tissue development. In the absence of catch-up growth before weaning, DEX and UN IUGR pups both presented basal hyperglycaemia, decreased glucose tolerance, and pancreatic islet atrophy. Other early metabolic defects were model-specific: DEX pups presented decreased insulin sensitivity whereas UN pups exhibited lowered glucose-induced insulin secretion and more marked alterations in gene expression of pancreatic islet and adipose tissue development regulators. In conclusion, these results show that before any catch-up growth, IUGR rats present early physiologic, morphologic and transcriptomic defects, which can be considered as initial mechanistic basis of metabolic programming.

## Introduction

Adverse fetal environment alters organ development and leads to poor fetal growth, a phenomenon also called intrauterine growth restriction (IUGR). Clinically, IUGR is commonly associated with an increased incidence in perinatal mortality but also with an elevated risk to develop chronic metabolic diseases (such as obesity and type 2 diabetes) later in life, reflecting noxious “metabolic programming” [1]. To elucidate the mechanisms involved in this deleterious process, IUGR can be experimentally induced in rodents by placental blood supply restriction [2], hypoxia [3], maternal diabetes [4] and more commonly by different manipulations of the maternal diet such as protein or caloric restriction [5]–[10]. Special focus has been placed on the impact of the IUGR modeling regarding endocrine pancreas development due to its central function in the control of glucose homeostasis [11]. Maternal low caloric diet decreases beta-cell neogenesis in relation to elevated glucocorticoid levels [12]. Indeed, excessive fetal exposure to glucocorticoids appears to be a common mechanism by which inadequate intrauterine environment leads to deleterious pancreatic islet development resulting in later metabolic disturbances. *In vitro* treatment of pancreatic buds with dexamethasone, a synthetic glucocorticoid, favors pancreatic differentiation into exocrine tissue and represses beta-cell differentiation [13], [14]. *In vivo*, dexamethasone exposure during fetal life, mimicking maternal stress, as well as pharmacological inhibition of the placental 11 beta-hydroxysteroid dehydrogenase type 2 (11β-HSD2), the enzyme protecting the fetus from maternal glucocorticoids, result in IUGR, decreased beta-cell pool and impaired glucose homeostasis [15], [16]. Inversely, mice harbouring a deletion of the glucocorticoid receptor in Pdx-1-expressing precursor cells present an elevation of beta-cell mass [13], [17]. Numerous studies dealing with IUGR and programming of metabolic diseases have focused on late-onset metabolic syndrome in IUGR pups [5]–[7], [18]–[20]. Less knowledge is currently available about pre-weaning metabolic defects in IUGR pups. Moreover, the causal link between early catch-up growth and metabolic programming remains elusive. In consequence, precocious metabolic investigations would allow to better understand the genesis of late onset metabolic syndrome. To this aim, we combined physiological, morphometric and transcriptomic approaches, focusing on endocrine pancreas and adipose tissue development during the early postnatal period. Rat IUGR pups were modeled through prenatal exposure to synthetic glucocorticoid (dams infused with dexamethasone, DEX) or through prenatal undernutrition (restricted pair-fed dams, UN). This set of experiments should allow better understanding precocious physiopathological processes involved in metabolic programming.

## Methods

### Animals and experimental design

All experimental protocols were approved by the “State of Geneva Veterinary Office” (Geneva, Switzerland). Female and male (275–325 g) Sprague-Dawley OFA rats (Charles River Laboratories, France) were used for breeding and housed under specific pathogen-free conditions in an environmentally-controlled clean room at the School of Medicine animal facilities (Medical Center University, University of Geneva). The animals were time mated (the day that sperm-positive smears were obtained was declared day 0 of gestation) and then caged individually before being assigned to one experimental group. In parallel a control group (CON) and two maternal models aimed to induce IUGR in progeny were used: the first model (DEX) consisted in dexamethasone (Sigma Aldrich, Buchs, Switzerland) exposure during the last week of gestation (infusion through an osmotic Alzet minipump delivering 100 µg/kg/day) and the second model (UN) consisted in maternal severe food restriction (gestational time-matched pair-feeding to 30% of normal food intake during the entire gestation). Body weights of gestating rats were monitored daily. At parturition (postnatal day 0, PND0), all pups were sexed, weighed and then nourished by their own mothers fed *ad libitum*. No reduction of litter size was performed, only excluding litters with extreme number of pups (<10 and >15). Post-delivery dam weights and food intake were monitored twice weekly and pup weights were monitored at PND7, 14 and 21. To avoid any sex confounding effect and interference in hormonal status, all investigations were performed on male pups only. One cohort of pups was sacrificed at PND7 (for hepatic gene expression and protein levels, pancreatic morphometry and histology, pancreatic islet gene expression analysis, pancreatic insulin content, corticosterone levels) and another cohort was sacrificed at PND21 (for white and brown adipose tissue investigations and leptin levels). Another independent cohort of animals was used at PND21 for a glucose tolerance test (GTT) and the same cohort was used after one week of recovery at PND28 for an insulin tolerance test (ITT). For physiological tests (GTT, ITT, insulin content), morphometric analyses (pancreatic islet size and number) and transcriptomic measures (gene expression in liver/pancreatic islets/adipose tissue), we studied pups originating from three independent litters in each group to take into account the intra-group variability. Animals in each group were randomly chosen but their weights were checked to best represent the mean of each group, excluding outliers.

### Histology and morphometry of pancreas

At PND7, pancreases of 5 to 6 rats in each group were fixed in Bouin's solution, embedded in paraffin and cut (5 µm sections). For morphometry, three sections of each pancreas were stained with aldehyde fuchsine before being analysed with a personalized program in MetaMorph® Imaging System (Meta Imaging Software, Molecular Devices Corporation, Pennsylvania, U.S.A). This program determines the total area of pancreatic tissue, the total area of Langerhans islets, the number of islets and their size. For number and size determination, only islets larger than 300 µm^2^ were used for calculations. For the immunohistochemical staining of insulin and glucagon positive cells, the sections were deparaffined with xylene and rehydrated through graded alcohols before permeabilisation (PBS-Triton 0.1%) and antigen blockade (PBS-BSA 5%). Sections were then incubated 2 h at room temperature with a solution containing a 1∶500 mouse monoclonal insulin antibody (Sigma Aldrich, Buchs, Switzerland) and a 1∶500 rabbit polyclonal glucagon antibody (Millipore, Billerica, MA, U.S.A). Sections were washed and incubated 45 min with secondary fluorescent antibodies AlexaFluor 488 goat anti-mouse and 555 goat anti-rabbit (respectively for insulin and glucagon). Images were taken using the Mirax system or the axiocam system (Carl Zeiss MicroImaging GmbH, Germany).

### Islet isolation

At PND7, pancreases were immediately excised and cut into small pieces in Hanks solution before transfer into 5 mL of Hanks-collagenase type V solution (Sigma Aldrich, Buchs, Switzerland) incubated in a 37°C water bath for 7 minutes. Digestion was stopped by addition of Hanks/BSA on ice and the pancreatic tissue was centrifuged several times before undigested fragments were carefully removed. After washing with Hanks solution, the tissue was concentrated into a pellet which was resuspended on a Histopaque® 1077 gradient (Sigma Aldrich, Buchs, Switzerland). After centrifugation at 2500 rpm for 20 minutes, islets were harvested from the interface between the layers, washed and finally concentrated into pellets and immediately used for RNA extraction.

### Gene expression

Total RNA from PND7 rat islets was extracted using the RNeasy Mini Kit® according to the manufacturer's protocol (Qiagen, Basel, Switzerland) and total RNA from PND7 liver, PND21 epididymal white adipose tissue (eWAT) and PND21 interscapular brown adipose tissue (iBAT) were prepared using the Trizol reagent (Invitrogen, Basel, Switzerland), according to manufacturer's instructions. One microgram of pancreatic islets total RNA and five micrograms of liver, eWAT and iBAT total RNA were reverse-transcribed using 800 units of Moloney murine leukemia virus reverse transcriptase (Invitrogen, Basel, Switzerland), in the presence of 0.3 units/µl RNAsin (Promega Corp, Madison, WI), 7.5 µM of random primers (oligo(dN)6), 1.2 mM dNTP and 12 µM of DTT. The expression of the cDNAs of interest were determined by quantitative real-time PCR using an ABI 7000 Sequence Detection System (Applera Europe, Rotkreuz, Switzerland) and were normalized using the housekeeping genes Ppia (cyclophilin) and Rplp0 (36B4). PCR products were quantified using the SYBR Green Core Reagent kit (Applera Europe, Rotkreuz, Switzerland) and results were expressed in arbitrary units (A.U) relative to the mean value of the control group. Primers sequences designed using the Primer Express software (Applera Europe, Rotkreuz, Switzerland) are listed in Table 1.

**Table 1:** PCR primers for quantitative real-time PCR.

Tissue	Gene	Forward	Reverse
**Liver**	**Pck1**	5′-ACAACTGTTGGCTGGCTCTCA-3′	5′-GGTAATGATGACCGTCTTGCTTTC-3′
**Liver**	**G6pc**	5′-ATCTACCTTGCGGCTCACTTTC-3′	5′-AAGTTTCAGCCAAGCAATGC-3′
**Islets**	**Foxa2**	5′-CATCCGACTGGAGCAGCTACT-3′	5′-CCCAGGCTGGCGTTCAT-3′
**Islets**	**Pdx1**	5′-CCGCGTTCATCTCCCTTT-3′	5′-CTCCTGCCCACTGGCTTTT-3′
**Islets**	**Onecut1**	5′-TCGGCGCTCCGCTTAG-3′	5′-CCTTCCCGTGTTCTTGCTCTT-3′
**Islets**	**Neurog3**	5′-AGAACTAGGATGGCGCCTCAT-3′	5′-CCGGCAAAAGGTTGTTGTGT-3′
**Islets**	**Nkx6-1**	5′-CCCCCATCAAGGATCCATTT-3′	5′-CTGCTGGCCGGAGAATGT-3′
**Islets**	**Pax6**	5′-CCAACGACAATATACCCAGTGTGT-3′	5′-GCGCCCATCTGTTGCTTTT-3′
**Islets**	**Mafa**	5′-CATTCTGGAGAGCGAGAAGTG-3′	5′-TTTCTCCTTGTACAGGTCCCG-3′
**Islets**	**Ins1**	5′-CAGCACCTTTGTGGTCCTCA-3′	5′-CCCACACACCAGGTACAGAGC-3′
**Islets**	**Gcg**	5′-CGCCGTGCTCAAGATTTTGT-3′	5′-CCGGTTCCTCTTGGTGTTCAT-3′
**WAT**	**Gata2**	5′-AATCGGCCGCTCATCAAG-3′	5′-TCGTCTGACAATTTGCACAACA-3′
**WAT**	**Dlk1**	5′-CTGCACTGACCCCATTTGTCT-3′	5′-TTCCCCCGGTTTGTCACA-3′
**WAT**	**Egr2**	5′-TGGCTGGAGATGGCATGAT-3′	5′-CTACTCGGATATGGGAGATCCAA-3′
**WAT**	**Klf5**	5′-GTCCGATACAACAGAAGGAGTAACC-3′	5′-ACTTTTGTGCAACCATCATAATCAC-3′
**WAT**	**Cebpa**	5′-AGTTGACCAGTGACAATGACCG-3′	5′-TCAGGCAGCTGGCGGAAGAT-3′
**WAT**	**Pparg**	5′-CTGACCCAATGGTTGCTGATTAC-3′	5′-GGACGCAGGCTCTACTTTGATC-3′
**WAT**	**Srebf1**	5′-CATCGACTACATCCGCTTCTTACA-3′	5′-GTCTTTCAGTGATTTGCTTTTGTGA-3′
**WAT**	**Lep**	5′-ATTTCACACACGCAGTCGGTAT-3′	5′-CCCGGGAATGAAGTCCAAA-3′
**WAT**	**Adipoq**	5′-AAGGGAGACGCAGGTGTTCTT-3′	5′-CCCTTCCGCTCCTGTCATT-3′
**WAT**	**Tnf**	5′-GACCCTCACACTCAGATCATCTTCT-3′	5′-TCCGCTTGGTGGTTTGCTA-3′
**WAT**	**Il6**	5′-ATATGTTCTCAGGGAGATCTTGGAA-3′	5′-TGCATCATCGCTGTTCATACAA-3′
**WAT**	**Il1rn**	5′-CAGCTGGAGGAGGTTAACATCAC-3′	5′-TCTCGGAGCGGATGAAGGTA-3′
**BAT**	**Ucp1**	5′-TGGGACCTACAATGCTTACAGAGTT-3′	5′-ATTAGATTAGGAGTCGTCCCTTTCC-3′
**BAT**	**Ppargc1a**	5′-CTGCCATTGTTAAGACCGAGAA-3′	5′-AGGGACGTCTTTGTGGCTTTT-3′
**BAT**	**Adrb3**	5′-GCCGTGGACCGGAAGAG-3′	5′-TAGAACTGTTGAGCGGTGAGTTCT-3′
**Housekeeping**	**Ppia**	5′-TCACCATCTCCGACTGTGGA-3′	5′-AAATGCCCGCAAGTCAAAGA-3′
**Housekeeping**	**Rplp0**	5′-TTCCCACTGGCTGAAAAGGT-3′	5′-CGCAGCCGCAAATGC-3′

### Western blot

Liver proteins were extracted from four rats in each group at 7 days of age and were subjected to reducing SDS-PAGE using 10% Tris gels. Proteins were then electroblotted from the gels on Nitrocellulose membranes (Amersham Hybond, GE Healthcare, Glattbrugg) and probed with a PEPCK2 primary antibody (1∶1000; Cell Signaling, Beverly, MA, USA) or a G6Pase primary antibody (1∶6000, as previously reported [21]), followed by rabbit anti-rat horseradish peroxidase-conjugated secondary antibody. ECL mediated by horseradish peroxidase was developed with the ECL kit and detected with Amersham hyperfilm (Amersham ECL Plus Western Blotting Detection Reagents, GE Healthcare, Glattbrugg).

### Pancreatic insulin content

At PND7, total pancreases were removed, frozen in liquid nitrogen and stored at −80°C. Tissues were later pulverized, resuspended in cold acid ethanol, and left at 4°C for 48 h, with sonication every 24 h. Insulin content in the acid ethanol supernatant were determined with a rat insulin RIA kit (Linco, Labodia, Yens, Switzerland).

### Glucose tolerance test and insulin tolerance test

For the glucose tolerance test, 21-day-old rats of each group were removed from their mother 5 h before receiving an i.p. injection containing 2 mg glucose (Sigma Aldrich, Buchs, Switzerland) per g of body weight. For the insulin tolerance test 7 days later, at PND28, the same rats were fasted for 3 h before receiving an i.p. injection containing 0.5 mU human recombinant insulin (Actrapid, Novo Nordisk, Bagsvaerd, Denmark) per g of body weight. Blood samples were collected by tail puncture for immediate glycaemia measurement using a glucometer (Glucotrend Premium, Roche Diagnostics, Rotkreuz, Switzerland).

### Corticosterone, leptin, insulin and C-peptide levels

Blood samples were collected through tail puncture. Plasmas were recuperated and frozen for later dosage. Corticosterone and leptin levels were assessed in gestating dams respectively by immunoassay (Corticosterone AC-4F1, IDS, Boldon, UK), and radioimmunoassay (Linco Research, St Charles, MO, U.S.A). Insulin and C-peptide levels were assessed in pups using the 10–1250 rat insulin or 10–1251 rat ultrasensitive insulin and 10–1172 rat C-peptide Elisa assays (Mercodia, Uppsala, Sweden).

### Statistics

Results are expressed as mean ± SEM. The unpaired Student's t-test and repeated-measures one-way analysis of variance (ANOVA) were used for comparison between groups of rats when appropriate. These tests were performed with SYSTAT 10.01 (SPSS, Chicago, IL). A p value of <0.05 was considered statistically significant.

## Results

### Maternal weights, leptin and corticosterone levels

**Figure 1:**
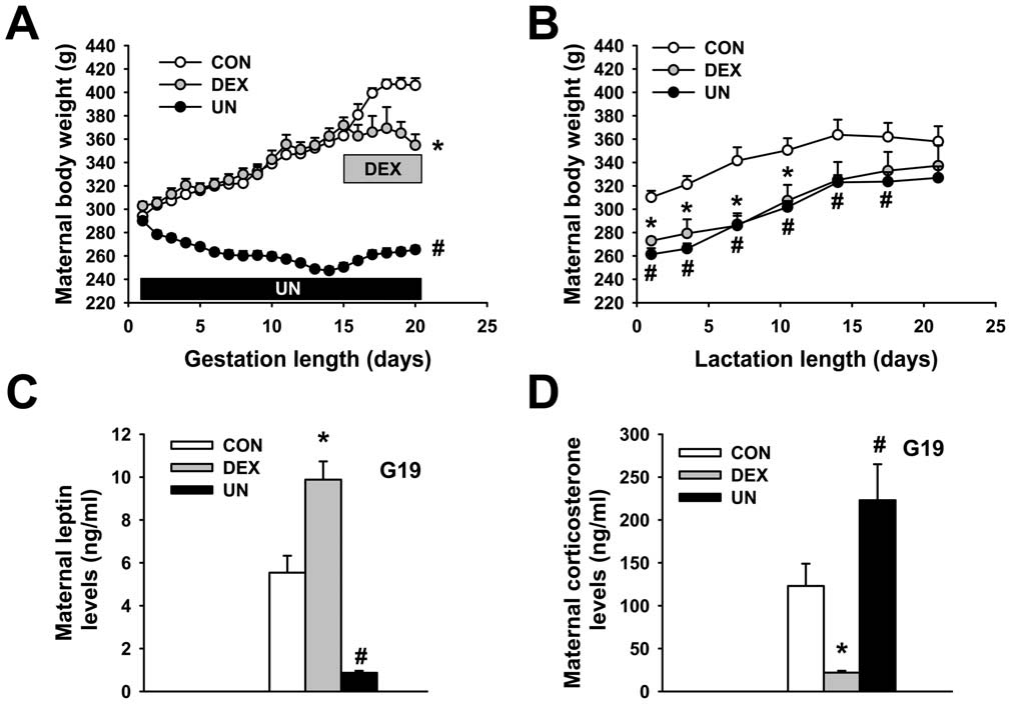
The effects of dexamethasone exposure and undernutrition on prenatal and postnatal maternal weight. Body weight curve during gestation (A) and post-delivery during lactation (B), circulating leptin levels (C) and circulating corticosterone levels (D) of control dams (CON), dexamethasone-exposed dams (DEX) and undernourrished dams (UN). Circulating leptin and corticosterone levels were measured at gestational day 19 (G19). N = 12–15 gestating rats per group, * p≤0.05 for DEX vs. CON group and # p≤0.05 for UN vs. CON group for panel A. N = 5 gestating rats per group, * p≤0.05 for DEX vs. CON group and # p≤0.05 for UN vs. CON group for panel B. N = 5 gestating rats per group, * p≤0.01 for DEX vs. CON group and # p≤0.002 for UN vs. CON group for panel C. N = 7–9 gestating rats per group, * p≤0.001 for DEX vs. CON group and # p≤0.05 for UN vs. CON group for panel D.

Gestation of control dams (CON) was monitored in parallel to those of dams exposed to dexamethasone (DEX) or undernutrition (UN). Total weight gain during gestation of CON dams was more than 100 g (Fig. 1A). In contrast, DEX dams exhibit an arrest in b.w. gain immediately after the beginning of infusion and UN dams lost approximately 25 g as compared to initial weight on gestational day 1 (Fig. 1A). Post-delivery body weight of DEX and UN dams remained lower than CON dams during lactation (Fig. 1B) but all groups consumed the same amount of food (data not shown). Gestational circulating leptin levels were increased in DEX dams (9.9±0.8 ng/ml, p<0.01), in agreement with the stimulatory effect of glucocorticoids on leptin production [22], [23], and decreased in UN dams (0.9±0.1 ng/ml, p<0.002) when compared to CON dams (5.5±0.8 ng/ml), probably reflecting the massive loss of fat used to counteract undernutrition (Fig. 1C). In the DEX dams, endogenous corticosterone levels were decreased to low levels (22±2 ng/ml, p<0.001), due to the negative feedback of the exogenous corticoid administration on endogenous production, whereas UN dams displayed two-fold higher corticosterone levels (223±42 ng/ml, p<0.05) than CON dams (123±26 ng/ml) (Fig. 1D), demonstrating a major metabolic stress.

### Perinatal body weight (b.w.) in IUGR pups

Neither litter size (13.0±1.2 pups/litter in CON group; 14.2±0.7 in DEX goup, p = 0.42; 12.5±0.6 in UN group, p = 0.73) nor sex ratio of born pups (47±5% of males in CON group; 54±3% in DEX group, p = 0.28; 47±4% in UN group, p = 0.98) were significantly different between the IUGR groups and the control group in this set of experiments (data not shown).

**Figure 2:**
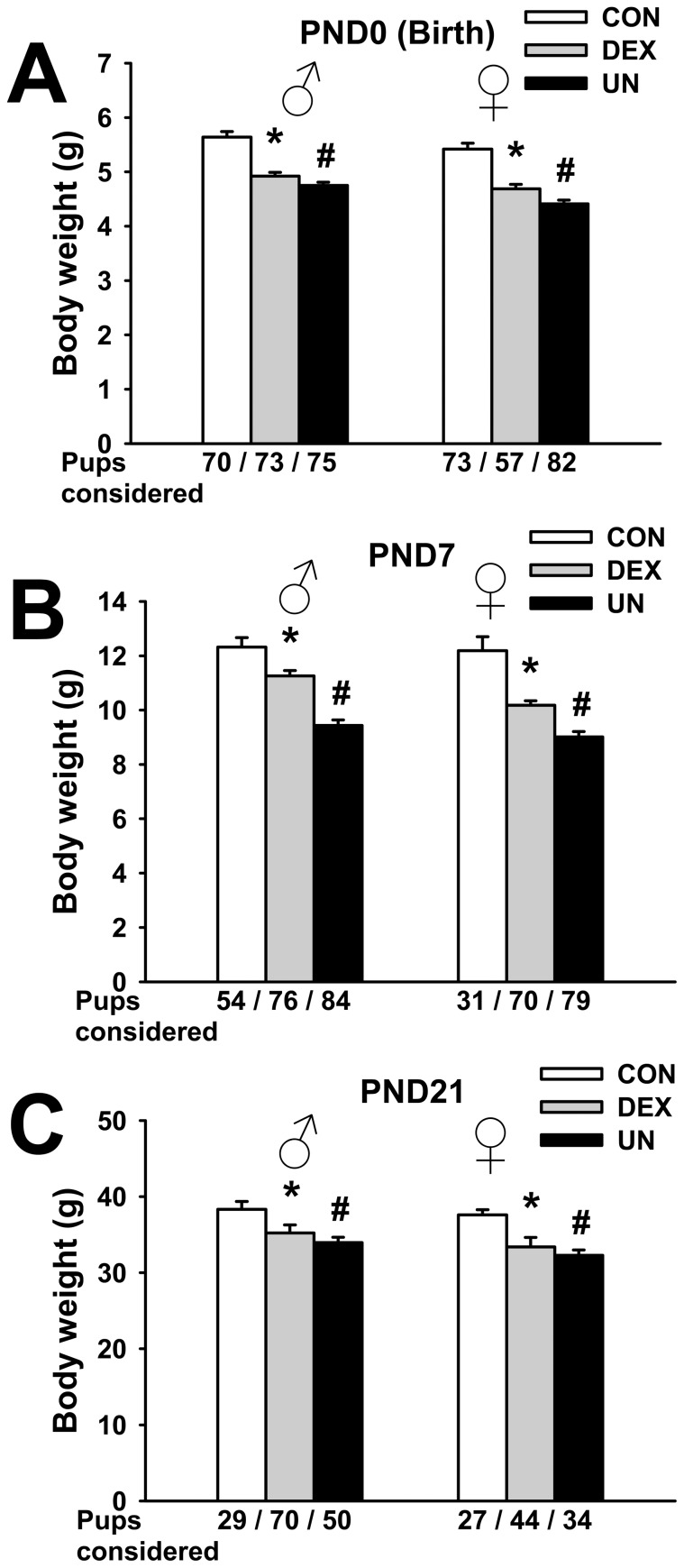
Postnatal body weight in IUGR pups. Body weight at birth (postnatal day 0, PND0) (A), at PND7 (B) and at PND21 (C) of male and female control pups (CON), prenatally dexamethasone-exposed pups (DEX) and prenatally undernourrished pups (UN). Number of pups considered is indicated below each corresponding bar. * p≤0.0001 for DEX vs. CON group and # p≤0.0001 for UN vs. CON group (panel A), * p≤0.005 for DEX vs. CON group and # p≤0.0001 for UN vs. CON group (panel B), * p≤0.01 for DEX vs. CON group and # p≤0.001 for UN vs. CON group (panel C).

As expected, birth b.w. (postnatal day 0, PND0) of pups exposed *in utero* to maternal dexamethasone or undernutrition were significantly reduced. In fact, both male (4.92±0.07 g, p<0.0001) and female (4.69±0.08 g, p<0.0001) DEX pups, as well as male (4.75±0.06 g, p<0.0001) and female (4.41±0.07 g, p<0.0001) UN pups were lighter compared to male (5.64±0.10 g) and female (5.42±0.11 g) CON pups (Fig. 2A), respectively. In consequence, without taking the slight sex difference into consideration, maternal dexamethasone and undernutrition exposure induced approximately 13% and 17% limitation of intrauterine growth, respectively. No major catch-up was observed during the first week of life, since at PND7, DEX and UN pup remained 13% (p<0.005) and 24% (p<0.0001) significantly lighter than CON pups (Fig. 2B), respectively. At PND7, circulating corticosterone levels of DEX pups (6.9±1.5 ng/ml, p = 0.77) were not different to those of CON pups (6.3±0.7 ng/ml) whereas corticosterone levels of UN pups were increased (14.6±2.7 ng/ml, p<0.02) (data not shown). Finally, at the end of the suckling period (PND21), DEX and UN pups were 10% (p<0.01) and 13% (p<0.0001) lighter than CON pups (Fig. 2C), respectively.

These results indicate that both DEX and UN IUGR pups failed to recover a normal b.w. at weaning, excluding spontaneous early postnatal catch-up. To avoid any sex and hormonal confounding interference, all the following results were obtained with male pups only.

### Early glucose homeostasis in IUGR pups

**Figure 3:**
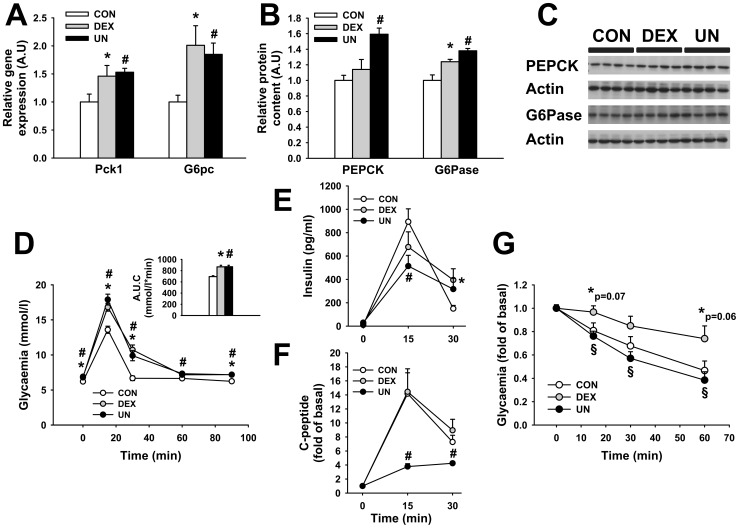
Glucose homeostasis in IUGR pups. Phosphoenolpyruvate carboxykinase (Pck1) and glucose-6-phosphatase (G6pc) mRNA (A) and protein (B and C) levels in liver at PND7, circulating glucose (D), insulin (E) and C-peptide (F) levels during a GTT at PND21, relative glycaemia during an ITT at PND28 (G) of male control pups (CON), prenatally dexamethasone-exposed pups (DEX) and prenatally undernourrished pups (UN). N = 6 male rats per group for panel A, N = 4 male rats per group for panel B and C, N = 12–15 male rats per group for panel D and G, N = 10–12 male rats per group for panel E and F. For all panel, * p≤0.05 or indicated value for DEX vs. CON group, # p≤0.05 for UN vs. CON group and § p≤0.05 for DEX vs. UN group.

The hepatic gene expression of phosphoenolpyruvate carboxykinase (Pck1) and glucose-6-phosphatase (G6pc) were increased by 46±19% (p<0.05) and 101±35% (p<0.05) in DEX pups and by 53±7% (p<0.05) and 85±20% (p<0.05) in UN pups, compared to CON pups at PND7 (Fig. 3A). The corresponding protein levels were also increased; PEPCK by 59±7% (p<0.002) in UN pups and G6Pase by 24±3% (p<0.03) in DEX and by 38±3% (p<0.01) in UN pups (Fig. 3B and 3C), suggesting a precocious elevation in gluconeogenesis and liver glucose output in these two IUGR rat models. This early defect in the control of glucose homeostasis was substantiated by elevated basal glycaemia (observed before weaning, at PND21, suckling-out), in the DEX (6.9±0.1 mmol/l, p<0.005) and the UN pups (6.8±0.1 mmol/l, p<0.02) when compared to the CON pups (6.2±0.2 mmol/l) (Fig. 3D, first time point). Moreover, the glycaemia during a glucose tolerance test (GTT) was also increased at the different time points in DEX and UN pups compared to CON pups (Fig. 3D), suggesting an attenuated glucose tolerance in IUGR pups as soon as PND21. This observation was confirmed by increased area under the curve (AUC) for the entire glycaemia excursion in DEX pups (868±27 mmol/L*min, p<0.0001) and UN pups (870±26 mmol/L*min, p<0.0001) compared to CON pups (693±16 mmol/L*min) (Fig. 3D, insert panel). Fasting insulin levels were undetectable in almost all pups from the three experimental groups at PND21 (Fig. 3E, first time point). Insulin secretion was decreased 15 minutes after glucose administration in UN (0.51±0.09 ng/mL, p<0.02) compared to CON pups (0.89±0.11 ng/mL) and increased 30 minutes after glucose administration in DEX pups (Fig. 3E). Fasting C-peptide levels were unchanged in DEX (260±40 pmol/L, p = 0.23) and increased in UN (511±40 pmol/L, p<0.001) compared to CON pups (203±23 pmol/L). Fold of basal C-peptide secretion was reduced 15 and 30 minutes following glucose administration in UN but not in DEX pups (Fig. 3F). To assess the global body sensitivity to insulin action, an insulin tolerance test (ITT) was performed on the same animal groups, one week later. At PND28, after 7 days of recovery and after a 3h-fast, the drops of glycaemia (Fig. 3G) in response to insulin administration were attenuated in DEX pups compared to UN and CON pups, suggesting a reduction in insulin sensitivity in relation to prenatal dexamethasone but not to undernutrition.

These results demonstrate that both DEX and UN pups present an early increase in gluconeogenesis and glucose intolerance. UN pups exhibit a decrease in glucose-induced insulin secretion whereas DEX pups exhibit a precocious decrease in insulin sensitivity.

### Early endocrine pancreatic morphology in IUGR pups

**Figure 4:**
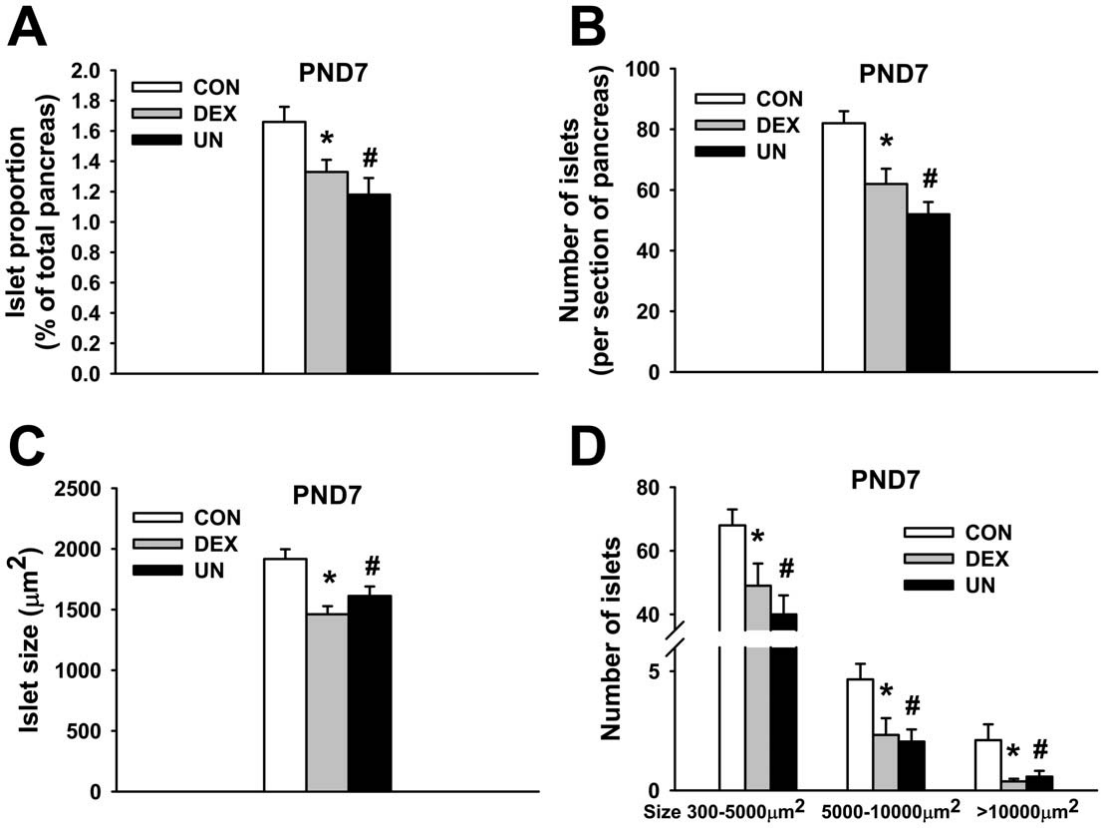
Morphological analysis of endocrine pancreas in IUGR pups. Islet area reported to whole pancreas area (A), number of islets per section (B), mean islet size expressed in µm^2^ (C) and number of small (300–5000µm^2^), medium (5000–10000µm^2^) and large (>10000µm^2^) islets per section (D) of male control pups (CON), prenatally dexamethasone-exposed pups (DEX) and prenatally undernourrished pups (UN). For all panel, N = 5–6 male rats per group, 3 pancreatic sections per rat, * p≤0.02 for DEX vs. CON group, # p≤0.005 for UN vs. CON group (A), * p≤0.005 for DEX vs. CON group, # p≤0.001 for UN vs. CON group (B), * p≤0.0001 for DEX vs. CON group, # p≤0.01 for UN vs. CON group (C), * p≤0.05 for DEX vs. CON group, # p≤0.05 for UN vs. CON group (D).

In order to evaluate the impact of IUGR modelling on endocrine pancreas development, morphometric pancreatic analysis were performed in DEX and UN pups at PND7 (Fig. 4). The pancreatic endocrine area was not modified in IUGR pups when reported to b.w. (data not shown) but the ratio of endocrine to whole pancreas area was significantly reduced both in DEX (1.33±0.08%, p<0.03) and UN (1.18±0.11%, p<0.005) pups compared to CON pups (1.66±0.10%) (Fig. 4A). The reduction of endocrine pancreas development was related to a concomitant decrease in the number of islets (62±5 islets/section, p<0.01 for DEX and 52±4, p<0.0001 for UN pups compared to 82±4 for CON pups) (Fig. 4B) and in the mean islet size (1462±66 µm^2^, p<0.0001 for DEX and 1612±79 µm^2^, p<0.02 for UN compared to 1917±80 μm^2^ for CON pups) (Fig. 4C). Quantitatively, the number of small islets (300–5000 µm^2^), medium islets (5000–10000 µm^2^) and large islets (>10000 µm^2^) per pancreatic section was reduced in DEX and UN pups compared to CON pups in each size category (Fig. 4D). Likewise, when expressed relatively to pancreatic surface unit, the number of small islets (6.88±0.37/mm^2^, p = 0.36 for DEX and 5.62±0.19/mm^2^, p<0.001 for UN pups), medium islets (0.30±0.06/mm^2^, p<0.03 for DEX and 0.27±0.05/mm^2^, p<0.01 for UN pups) and large islets (0.05±0.01/mm^2^, p<0.02 for DEX and 0.08±0.03/mm^2^, p = 0.08 for UN pups) remained globally lower in IUGR pups compared to CON pups (7.37±0.3 small islets/mm^2^, 0.53±0.06 medium islets/mm^2^ and 0.21±0.06 large islets/mm^2^) (data not shown).

These results demonstrate that both DEX and UN pups presented an altered endocrine pancreatic development due to a concomitant reduction in the size and number of Langerhans islets.

### Early pancreatic hormone production in IUGR pups

To extend these morphological investigations, we first studied the transcriptional changes in pancreatic islets isolated from DEX and UN pups at PND7 with a special focus on transcription factors known to orchestrate the beta- and alpha-cell differentiation process.

**Figure 5:**
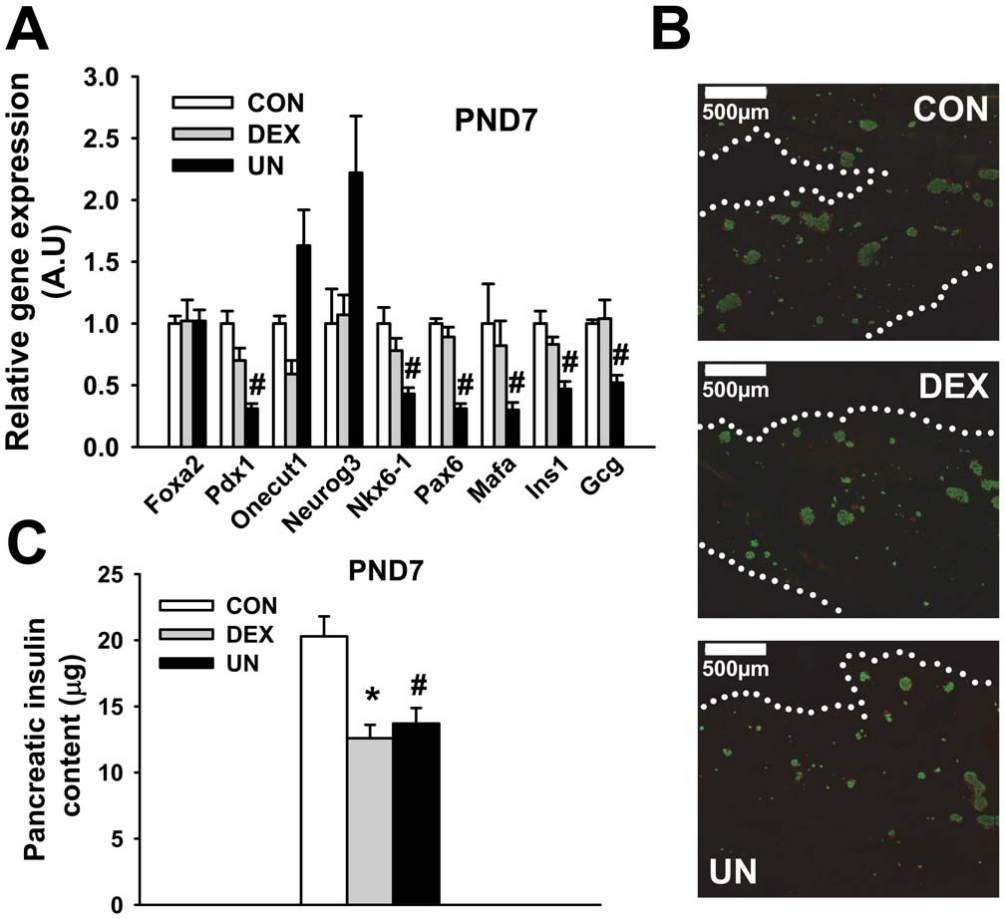
Endocrine pancreas hormone production in IUGR pups. mRNA levels of transcription factors and hormones in pancreatic islets (A), pancreatic immunolabelling of insulin (green) and glucagon (red) (B), pancreatic insulin content (C) of male control pups (CON), prenatally dexamethasone-exposed pups (DEX) and prenatally undernourrished pups (UN) at PND7. N = 3–8 pools of 3 male rats per group (panel A), N = 5–6 male rats per group (panel C). * p≤0.05 for DEX vs. CON group, # p≤0.05 for UN vs. CON group for all panels. Dotted white lines indicate the delimitation of the pancreatic tissue in panel B. Note the lower density and reduced size of islets from DEX and UN pups in panel B.

Whereas no change in HNF-3β (Foxa2) gene expression was observed in pancreatic islets of IUGR pups (Fig. 5A), Pdx1, Nkx6-1, Pax6 and Mafa gene expression were all specifically decreased in UN islets compared to CON islets (Fig. 5A). A trend to increased gene expression for Neurogenin3 (Neurog3) and HNF6 (Onecut1), its upstream regulator, was also observed in UN islets compared to CON islets (Fig. 5A), suggesting a selectivity in these transcriptional modifications. In accordance with the gene repression of the main endocrine transcription factor, we measured significant decrease in both insulin (Ins1) and glucagon (Gcg) mRNA levels in islets from UN pups, indicating an alteration in alpha- and beta-cell activity following prenatal undernutrition exposure. The mRNA levels of the glucose transporter 2 (Glut2), Kir6.2, a subunit of the K_ATP_ channel, and the enzyme glucokinase, all classical beta-cell marker genes, were also reduced by 60% to 65% in UN islets (data not shown). Transcriptional changes were less pronounced in islets from DEX pups (Fig. 5A), in line with unaltered secretory capacity. Immunolabelling of insulin and glucagon positive cells (Fig. 5B) showed no visual evidence of disorganization in IUGR islets (Fig. 5B). Glucagon positive cells represented 21±3% of the islet area in both CON and DEX pups and 26±1% in UN pups (p = 0.10 vs CON), suggesting a downward trend in beta-cell/alpha-cell proportion in UN pups. Whole pancreas insulin content (12.6±1.0 μg for DEX, 13.7±1.2 μg for UN, p<0.01, compared to 20.3±1.5 μg for CON pups) (Fig. 5C) corroborates the morphological alterations previously depicted in Fig. 4.

Taken together, these results demonstrate a perinatal developmental defect of the pancreatic endocrine cells in IUGR pups.

### Early adipose tissue development in IUGR pups

**Figure 6:**
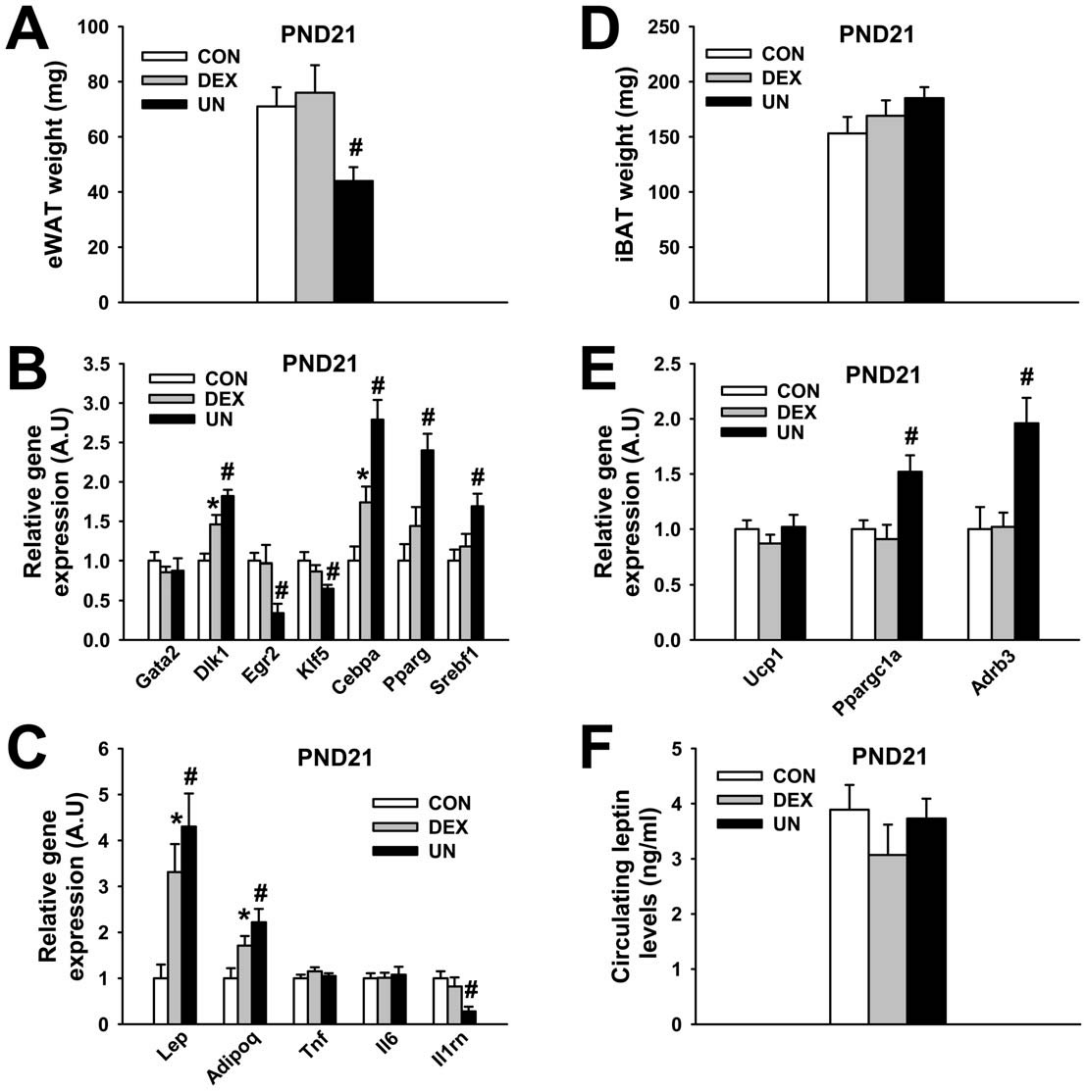
Adipose tissue deposition and transcriptional changes in IUGR pups at weaning. Weight of epididymal white adipose tissue (eWAT) (A), mRNA levels of transcription factors in eWAT (B), mRNA levels of adipokines in eWAT (C), weight of interscapular brown adipose tissue (iBAT) (D), mRNA levels of markers in iBAT (E), circulating leptin levels (F) in male control pups (CON), prenatally dexamethasone-exposed pups (DEX) and prenatally undernourrished pups (UN) at PND21. N = 10 (panel A–E), N = 5 (panel F) male rats per group, * p≤0.05 for DEX vs. CON group, # p≤0.05 for UN vs. CON group for all panels.

The early suckling period constitutes the most active period for adipose tissue development in rats [24]. We investigated adipose tissue deposition at PND21 in DEX and UN IUGR pups. As shown in Fig. 6A, DEX pups present the same amount of epididymal white adipose tissue (eWAT) (77±10 mg, p = 0.65) as CON pups (71±7 mg) whereas UN pups show reduced eWAT accretion (44±5 mg, p<0.01) (Fig. 6A). These observations remained true when eWAT mass was reported to b.w. (0.18±0.01%, p = 0.86 for DEX and 0.11±0.01%, p<0.0001 for UN compared to 0.18±0.01% for CON pups). No major change was observed in DEX and UN white adipocyte size and morphology (data not shown) but the analysis of gene expression revealed several transcriptional modifications, mainly in UN pups. Among preadipocyte markers, Pref-1 (Dlk1) was over-expressed in eWAT from DEX and UN pups (Fig. 6B). Gene expression of Krox-20 (Egr2) and KLF5 (Klf5), early stage adipogenesis regulators, were both reduced in eWAT from UN pups compared to CON pups (Fig. 6B). Inversely, gene expression of C/EBP-α (Cebpa), PPAR-γ (Pparg) and SREBP-1c (Srebf1), more late stage adipogenesis regulators, were all increased in eWAT from UN compared to eWAT from CON pups (Fig. 6B). Among adipokines, gene expression of leptin (Lep) and adiponectin (Adipoq) were elevated in eWAT from DEX and UN pups whereas gene expression of TNF-α (Tnf) and IL-6 (Il6) remained unchanged and gene expression of IL-1Ra (Il1rn) was decreased in eWAT from UN pups (Fig. 6C). No statistical change in the amount of interscapular brown adipose tissue (iBAT) was observed in DEX (169±15 mg, p = 0.46) and UN (185±10 mg, p = 0.09) IUGR pups compared to CON pups (153±15 mg) (Fig. 6D). iBAT mass reported to b.w. remained unchanged in DEX pups (0.41±0.03%, p = 0.70) but was increased in UN pups (0.51±0.02%, p<0.01) when compared to CON pups (0.39±0.03%) (data not shown). No change in gene expression of UCP-1 (Ucp1) was observed in iBAT between the different groups but an over-expression of PGC-1α (Ppargc1a) and β3-adrenergic receptor (Adrb3) was detected in iBAT from UN pups compared to CON pups (Fig. 6E). Circulating leptin levels were not changed at PND21 in DEX (3.1±0.5 ng/ml, p = 0.28) and UN pups (3.7±0.4 ng/ml, p = 0.79) compared to CON pups (3.9±0.4 ng/ml) (Fig. 6F).

## Discussion

Our present study attempted to better describe and understand the early metabolic defects in IUGR rat pups, modeled either by prenatal exposure to synthetic glucocorticoid or through prenatal undernutrition. As expected, birth b.w. of male and female pups from DEX and UN dams were severely lowered (13 to 17%), mimicking the human situation in which IUGR is defined as a fetal weight below the 10th percentile for gestational age.

Early postnatal accelerated weight gain is clinically observed in IUGR newborns during infancy [25], [26]. This catch-up growth is considered as one major mechanism contributing to their predisposition to later develop metabolic disorders [27]–[30]. Thus, rodent models could be useful to dissociate the noxious effects of low birth weight per se to those of a subsequent catch-up growth. In our present study, both DEX and UN IUGR pups remained lighter than control pups until PND21 (weaning). Our post-weaning observations, outside the field of the present study, showed however that DEX rats remained permanently lighter than control rats, in line with a previous report [20], whereas UN rats recovered a normal b.w after 4–6 weeks of chow diet consumption without any hyperphagia (data not shown). The absence of early catch-up in our UN pups could be linked to 1) the stringency of prenatal maternal undernutrition used (moderate food restriction allows pups to recover normal b.w at weaning [31], [32] contrary to severe food restriction [8], [19]), 2) to poor nutrition provided by remaining underweight postpartum DEX and UN mothers nevertheless eating the same amount of food as CON and 3) to the strain of rats used (Sprague-Dawley rats exhibit a slightly delayed catch-up growth phase compared to Wistar rats [33]).

Therefore, we used a combination of physiological, morphometric and transcriptomic approaches to study pancreatic islet and adipose tissue development during the early postnatal period to confirm that DEX and UN IUGR rats remain prone to early metabolic defects independently of any catch-up growth.

We initially report that both DEX and UN pups present early elevation in circulating glucose levels at PND21, just before weaning. This basal hyperglycaemia is substantiated by an early increase in gluconeogenesis demonstrated by hepatic over-expression and increased protein content of G6Pase and PEPCK in DEX and UN pups as soon as PND7. This precocious elevation in gluconeogenesis seems permanent since it was also reported in adult offspring prenatally exposed to dexamethasone showing increase in PEPCK mRNA and activity in the liver [15]. Dexamethasone-induced programming of later hyperglycaemia was also previously demonstrated to be fetus-focused [34], sex-dependent [35] and transgenerational [36]. Mechanistically, this programming process involved elevated Hnf4a gene expression due to a shift from its fetal to adult promoter utilization occurring in the liver periportal zone [37]. We also presently report that DEX and UN pups show an early intolerance to glucose administration observable as soon as PND21. Once again, this early defect mirrors the decreased glucose utilization observed in adult offspring prenatally exposed to dexamethasone [35] or undernutrition [32], [38].

Additional causes for the early impaired glucose utilization, beyond shared elevation of gluconeogenesis, appeared to be model-specific for DEX and UN pups. In fact, DEX pups present early attenuated insulin sensitivity, assessed by ITT, compared to UN pups. ITT did not allow to decipher tissular origin of insulin resistance and is less sensitive than standard euglycaemic hyperinsulinemic clamps used in older IUGR rodents [2], [39], [40] but are technically unsuitable in frail IUGR pups. Nevertheless, our early observations corroborate data from older animals demonstrating both insulin resistance in prenatally dexamethasone exposed rats [41] and improved insulin sensitivity at adulthood in perinatally malnourished animals devoid of catch-up growth [42]. Taken together, our physiological measurements demonstrate that impaired glucose homeostasis, characteristic of metabolic programming, is already present around weaning in IUGR pups, before any sign of catch-up growth. At tissular level, development of the endocrine pancreas in UN pups seems differently affected than in DEX pups. In fact, if a reduction in number and size of pancreatic islets as well as in insulin content is observed in both models, in agreement with previous observations [9], [12], [43], [44], transcriptomic alterations of hormones and endocrine transcription factors were more marked in UN pups in line with their selective defects in glucose-induced insulin secretory capacity. Exacerbated glucocorticoid signaling during pancreas development, extensively investigated [13], [14], [17], could be only one of the cause of IUGR pancreatic islet impairment. Differences in IUGR pup islet development could also be linked to the prenatal availability of essential nutrients. In this way, maternal low protein supply induces an increase in beta-cell apoptosis in progeny and a decrease in beta-cell proliferation whereas low caloric diet decreases beta-cell neogenesis [12], in agreement with the transcriptional alterations in regulators of pancreatic endocrine cell lineages that we observed in UN pups. A developmental delay in maturation of beta-cell precursors could be a possible etiology for reduced beta-cell mass in UN rat pups as substantiated by downregulation of Mafa and mature markers of beta-cell lineage in UN islets.

We finally investigated the impact of IUGR modeling regarding pup adipose development at PND21 since the suckling period is critical concerning the ontogeny of this metabolic tissue in rodents [24]. First, we observed no change in eWAT deposition in DEX pups but a specific decrease in eWAT mass (even reported to b.w.) in UN pups compared to control pups. Interestingly, this reduction in adipose tissue deposition was accompanied by an increased gene expression of the preadipocyte marker Pref-1 (Dlk1) in eWAT from UN pups. This over-expression could reflect either an increase in the pool of preadipocytes already present in the eWAT from UN pups at weaning, providing a cellular basis to the obesity–prone status of UN pups at adulthood, or a developmental delay in a fraction of the preadipocyte pool. Early programming of a mature adipose tissue mass due to an adverse prenatal environment is still debated. Some reports refute a change in adipocyte proliferation [45] or glucose uptake [5] in offspring prenatally malnourished, suggesting that alterations in fat accretion is secondarily to other metabolic defects involving neuroendocrine or pancreatic functions. Conversely, other studies relate primary alterations in WAT of IUGR offspring. In fact, a global up-regulation of genes involved in nutrient metabolism and adipocyte differentiation [46], a higher precocious proliferative capacity [47] and a higher glucose uptake [48] were observed in adipocytes isolated from IUGR pups. Over-expression of C/EBP-α (Cebpa), PPAR-γ (Pparg), SREBP-1C (Srebf1), later stage adipogenesis regulators detected in eWAT from UN pups at PND21 are in line with up-regulation of PPAR-γ2 observed as soon as PND1 and upregulation of SREBP-1C observed at adulthood in male IUGR rats [49]. These results explain that primary changes in the adipogenesis program, prior to the development of obesity in IUGR pups, could be initiating mechanisms involved in the so-called thrifty phenotype programming.

Adipose tissue could play an important role in linking poor fetal growth to later onset of adult diseases not only through its own over-development, but also through its secretion of numerous hormones (adipokines) modulating the metabolism. Clinical data suggest that differential regulation of adipokines in IUGR children may influence the risk for development of chronic diseases later in life [50]. In our UN model, we observed that despite eWAT depot hypotrophy, circulating leptin levels were not altered which could be explained by the increased leptin gene expression observed. A previous study has shown that IUGR pups present increased plasma leptin levels at weaning [10], suggesting that disproportionate production of leptin relative to fat mass could play a role in the etiology of metabolic programming, as previously suggested by Vickers et al [19]. Among other adipokines investigated, the expression of adiponectin (Adipoq) was also elevated in eWAT from DEX and UN pups, possibly to enhance fatty acid use. Gene expression of pro-inflammatory cytokines such as TNF-α (Tnf) and IL-6 (Il6) remained unchanged, demonstrating that adipose transcriptional alterations in UN pups at PND21 were specific and not associated with an early inflammatory state. This observation was also confirmed by the down-expression in eWAT from UN pups of IL-1 receptor antagonist (Il1rn), an acute-phase adipokine intervening in the counterregulation of the inflammatory processes [51] and characterized as a good marker of adiposity [52], [53]. Finally, the slight relative increase in brown adipose tissue deposition observed in UN pups at weaning, associated with increased gene expression of PGC-1α (Ppargc1a) and β3-adrenoreceptor (Adrb3) could contribute to elevate thermogenesis and maintain body temperature more efficiently in these frail pups, in line with a recent report describing an over-expression of UCP1 associated with a white adipose tissue brown-like phenotype in perinatally food restricted pups [54].

In conclusion, we modeled IUGR rat pups through maternal dexamethasone or undernutrition exposure. In the absence of catch-up growth before weaning, DEX and UN pups presented some similar early metabolic defects, such as basal hyperglycaemia and decreased tolerance to glucose. Model-specific early metabolic defects were also observed, since DEX pups presented decreased insulin sensitivity whereas UN pups exhibited more marked defects in glucose-induced insulin secretion and gene expression of regulators of pancreatic islet and adipose tissue development which can be considered as mechanistic basis of the metabolic programming. Finally, the metabolic syndrome can be detected early in IUGR pups, independently of any catch-up growth, and with model-specific features.

## References

[1] Hales CN, Barker DJ, Clark PM, Cox LJ, Fall C, et al (1991) Fetal and infant growth and impaired glucose tolerance at age 64. BMJ 303: 1019–1022. 1954451 10.1136/bmj.303.6809.1019 PMC1671766

[2] Vuguin P, Raab E, Liu B, Barzilai N, Simmons R (2004) Hepatic insulin resistance precedes the development of diabetes in a model of intrauterine growth retardation. Diabetes 53: 2617–2622. 15448092 10.2337/diabetes.53.10.2617

[3] Tapanainen PJ, Bang P, Wilson K, Unterman TG, Vreman HJ, et al (1994) Maternal hypoxia as a model for intrauterine growth retardation: effects on insulin-like growth factors and their binding proteins. Pediatr Res 36: 152–158. 7526325 10.1203/00006450-199408000-00004

[4] Eriksson UJ, Jansson L (1984) Diabetes in pregnancy: decreased placental blood flow and disturbed fetal development in the rat. Pediatr Res 18: 735–738. 6472944 10.1203/00006450-198408000-00012

[5] Jimenez-Chillaron JC, Hernandez-Valencia M, Reamer C, Fisher S, Joszi A, et al (2005) Beta-cell secretory dysfunction in the pathogenesis of low birth weight-associated diabetes: a murine model. Diabetes 54: 702–711. 15734846 10.2337/diabetes.54.3.702

[6] Erhuma A, Salter AM, Sculley DV, Langley-Evans SC, Bennett AJ (2007) Prenatal exposure to a low-protein diet programs disordered regulation of lipid metabolism in the aging rat. American journal of physiology Endocrinology and metabolism 292: E1702–1714. 17299084 10.1152/ajpendo.00605.2006 PMC1890310

[7] Yura S, Itoh H, Sagawa N, Yamamoto H, Masuzaki H, et al (2005) Role of premature leptin surge in obesity resulting from intrauterine undernutrition. Cell metabolism 1: 371–378. 16054086 10.1016/j.cmet.2005.05.005

[8] Garofano A, Czernichow P, Breant B (1998) Postnatal somatic growth and insulin contents in moderate or severe intrauterine growth retardation in the rat. Biology of the neonate 73: 89–98. 9483301 10.1159/000013964

[9] Blondeau B, Avril I, Duchene B, Breant B (2002) Endocrine pancreas development is altered in foetuses from rats previously showing intra-uterine growth retardation in response to malnutrition. Diabetologia 45: 394–401. 11914745 10.1007/s00125-001-0767-4

[10] Desai M, Gayle D, Babu J, Ross MG (2005) Programmed obesity in intrauterine growth-restricted newborns: modulation by newborn nutrition. Am J Physiol Regul Integr Comp Physiol 288: R91–96. 15297266 10.1152/ajpregu.00340.2004

[11] Schwitzgebel VM, Somm E, Klee P (2009) Modeling intrauterine growth retardation in rodents: Impact on pancreas development and glucose homeostasis. Mol Cell Endocrinol 304: 78–83. 19433251 10.1016/j.mce.2009.02.019

[12] Reusens B, Remacle C (2006) Programming of the endocrine pancreas by the early nutritional environment. Int J Biochem Cell Biol 38: 913–922. 16337425 10.1016/j.biocel.2005.10.012

[13] Gesina E, Tronche F, Herrera P, Duchene B, Tales W, et al (2004) Dissecting the role of glucocorticoids on pancreas development. Diabetes 53: 2322–2329. 15331541 10.2337/diabetes.53.9.2322

[14] Shen CN, Seckl JR, Slack JM, Tosh D (2003) Glucocorticoids suppress beta-cell development and induce hepatic metaplasia in embryonic pancreas. The Biochemical journal 375: 41–50. 14509268 10.1042/bj20030140 PMC1223676

[15] Nyirenda MJ, Lindsay RS, Kenyon CJ, Burchell A, Seckl JR (1998) Glucocorticoid exposure in late gestation permanently programs rat hepatic phosphoenolpyruvate carboxykinase and glucocorticoid receptor expression and causes glucose intolerance in adult offspring. The Journal of clinical investigation 101: 2174–2181. 9593773 10.1172/JCI1567 PMC508805

[16] Lindsay RS, Lindsay RM, Waddell BJ, Seckl JR (1996) Prenatal glucocorticoid exposure leads to offspring hyperglycaemia in the rat: studies with the 11 beta-hydroxysteroid dehydrogenase inhibitor carbenoxolone. Diabetologia 39: 1299–1305. 8932995 10.1007/s001250050573

[17] Gesina E, Blondeau B, Milet A, Le Nin I, Duchene B, et al (2006) Glucocorticoid signalling affects pancreatic development through both direct and indirect effects. Diabetologia 49: 2939–2947. 17001468 10.1007/s00125-006-0449-3 PMC1885455

[18] Holemans K, Verhaeghe J, Dequeker J, Van Assche FA (1996) Insulin sensitivity in adult female rats subjected to malnutrition during the perinatal period. Journal of the Society for Gynecologic Investigation 3: 71–77. 8796811 10.1016/1071-5576(95)00046-1

[19] Vickers MH, Breier BH, Cutfield WS, Hofman PL, Gluckman PD (2000) Fetal origins of hyperphagia, obesity, and hypertension and postnatal amplification by hypercaloric nutrition. American journal of physiology Endocrinology and metabolism 279: E83–87. 10893326 10.1152/ajpendo.2000.279.1.E83

[20] Cleasby ME, Kelly PA, Walker BR, Seckl JR (2003) Programming of rat muscle and fat metabolism by in utero overexposure to glucocorticoids. Endocrinology 144: 999–1007. 12586777 10.1210/en.2002-220559

[21] Mutel E, Gautier-Stein A, Abdul-Wahed A, Amigo-Correig M, Zitoun C, et al (2011) Control of blood glucose in the absence of hepatic glucose production during prolonged fasting in mice: induction of renal and intestinal gluconeogenesis by glucagon. Diabetes 60: 3121–3131. 22013018 10.2337/db11-0571 PMC3219939

[22] Slieker LJ, Sloop KW, Surface PL, Kriauciunas A, LaQuier F, et al (1996) Regulation of expression of ob mRNA and protein by glucocorticoids and cAMP. The Journal of biological chemistry 271: 5301–5304. 8621378 10.1074/jbc.271.10.5301

[23] Sugden MC, Langdown ML, Munns MJ, Holness MJ (2001) Maternal glucocorticoid treatment modulates placental leptin and leptin receptor expression and materno-fetal leptin physiology during late pregnancy, and elicits hypertension associated with hyperleptinaemia in the early-growth-retarded adult offspring. European journal of endocrinology/European Federation of Endocrine Societies 145: 529–539. 10.1530/eje.0.1450529 11581014

[24] Pouteau E, Turner S, Aprikian O, Hellerstein M, Moser M, et al (2008) Time course and dynamics of adipose tissue development in obese and lean Zucker rat pups. Int J Obes (Lond) 32: 648–657. 18087263 10.1038/sj.ijo.0803787

[25] Ong KK, Ahmed ML, Emmett PM, Preece MA, Dunger DB (2000) Association between postnatal catch-up growth and obesity in childhood: prospective cohort study. Bmj 320: 967–971. 10753147 10.1136/bmj.320.7240.967 PMC27335

[26] McMillen IC, Robinson JS (2005) Developmental origins of the metabolic syndrome: prediction, plasticity, and programming. Physiol Rev 85: 571–633. 15788706 10.1152/physrev.00053.2003

[27] Soto N, Bazaes RA, Pena V, Salazar T, Avila A, et al (2003) Insulin sensitivity and secretion are related to catch-up growth in small-for-gestational-age infants at age 1 year: results from a prospective cohort. The Journal of clinical endocrinology and metabolism 88: 3645–3650. 12915649 10.1210/jc.2002-030031

[28] Hofman PL, Cutfield WS, Robinson EM, Bergman RN, Menon RK, et al (1997) Insulin resistance in short children with intrauterine growth retardation. The Journal of clinical endocrinology and metabolism 82: 402–406. 9024226 10.1210/jcem.82.2.3752

[29] Jaquet D, Gaboriau A, Czernichow P, Levy-Marchal C (2000) Insulin resistance early in adulthood in subjects born with intrauterine growth retardation. The Journal of clinical endocrinology and metabolism 85: 1401–1406. 10770173 10.1210/jcem.85.4.6544

[30] Eriksson JG, Forsen TJ, Osmond C, Barker DJ (2003) Pathways of infant and childhood growth that lead to type 2 diabetes. Diabetes care 26: 3006–3010. 14578231 10.2337/diacare.26.11.3006

[31] Ozaki T, Nishina H, Hanson MA, Poston L (2001) Dietary restriction in pregnant rats causes gender-related hypertension and vascular dysfunction in offspring. The Journal of physiology 530: 141–152. 11136866 10.1111/j.1469-7793.2001.0141m.x PMC2278385

[32] Shahkhalili Y, Moulin J, Zbinden I, Aprikian O, Mace K (2010) Comparison of two models of intrauterine growth restriction for early catch-up growth and later development of glucose intolerance and obesity in rats. American journal of physiology Regulatory, integrative and comparative physiology 298: R141–146. 10.1152/ajpregu.00128.2009 19889868

[33] Ellis-Hutchings RG, Zucker RM, Grey BE, Norwood J Jr, Richards JH, et al (2010) Altered health outcomes in adult offspring of Sprague Dawley and Wistar rats undernourished during early or late pregnancy. Birth Defects Res B Dev Reprod Toxicol 89: 396–407. 20973054 10.1002/bdrb.20265

[34] Nyirenda MJ, Welberg LA, Seckl JR (2001) Programming hyperglycaemia in the rat through prenatal exposure to glucocorticoids-fetal effect or maternal influence? The Journal of endocrinology 170: 653–660. 11524246 10.1677/joe.0.1700653

[35] O'Regan D, Kenyon CJ, Seckl JR, Holmes MC (2004) Glucocorticoid exposure in late gestation in the rat permanently programs gender-specific differences in adult cardiovascular and metabolic physiology. Am J Physiol Endocrinol Metab 287: E863–870. 15238353 10.1152/ajpendo.00137.2004

[36] Drake AJ, Walker BR, Seckl JR (2005) Intergenerational consequences of fetal programming by in utero exposure to glucocorticoids in rats. Am J Physiol Regul Integr Comp Physiol 288: R34–38. 15178540 10.1152/ajpregu.00106.2004

[37] Nyirenda MJ, Dean S, Lyons V, Chapman KE, Seckl JR (2006) Prenatal programming of hepatocyte nuclear factor 4alpha in the rat: A key mechanism in the ‘foetal origins of hyperglycaemia’? Diabetologia 49: 1412–1420. 16570165 10.1007/s00125-006-0188-5

[38] Bieswal F, Ahn MT, Reusens B, Holvoet P, Raes M, et al (2006) The importance of catch-up growth after early malnutrition for the programming of obesity in male rat. Obesity 14: 1330–1343. 16988075 10.1038/oby.2006.151

[39] Holness MJ (1996) Impact of early growth retardation on glucoregulatory control and insulin action in mature rats. The American journal of physiology 270: E946–954. 8764177 10.1152/ajpendo.1996.270.6.E946

[40] Raab EL, Vuguin PM, Stoffers DA, Simmons RA (2009) Neonatal exendin-4 treatment reduces oxidative stress and prevents hepatic insulin resistance in intrauterine growth-retarded rats. American journal of physiology Regulatory, integrative and comparative physiology 297: R1785–1794. 10.1152/ajpregu.00519.2009 PMC2803622 19846744

[41] Buhl ES, Neschen S, Yonemitsu S, Rossbacher J, Zhang D, et al (2007) Increased hypothalamic-pituitary-adrenal axis activity and hepatic insulin resistance in low-birth-weight rats. Am J Physiol Endocrinol Metab 293: E1451–1458. 17895287 10.1152/ajpendo.00356.2007 PMC2761595

[42] Lim K, Armitage JA, Stefanidis A, Oldfield BJ, Black MJ (2011) Intrauterine Growth Restriction in the Absence of Postnatal ‘Catch-Up’ Growth Leads to Improved Whole Body Insulin Sensitivity in Rat Offspring. Pediatr Res. 10.1203/PDR.0b013e31822a65a3 21885936

[43] Garofano A, Czernichow P, Breant B (1997) In utero undernutrition impairs rat beta-cell development. Diabetologia 40: 1231–1234. 9349607 10.1007/s001250050812

[44] Dumortier O, Blondeau B, Duvillie B, Reusens B, Breant B, et al (2007) Different mechanisms operating during different critical time-windows reduce rat fetal beta cell mass due to a maternal low-protein or low-energy diet. Diabetologia 50: 2495–2503. 17882398 10.1007/s00125-007-0811-0

[45] Bieswal F, Hay SM, McKinnon C, Reusens B, Cuignet M, et al (2004) Prenatal protein restriction does not affect the proliferation and differentiation of rat preadipocytes. J Nutr 134: 1493–1499. 15173417 10.1093/jn/134.6.1493

[46] Guan H, Arany E, van Beek JP, Chamson-Reig A, Thyssen S, et al (2005) Adipose tissue gene expression profiling reveals distinct molecular pathways that define visceral adiposity in offspring of maternal protein-restricted rats. American journal of physiology Endocrinology and metabolism 288: E663–673. 15562247 10.1152/ajpendo.00461.2004

[47] Bol VV, Reusens BM, Remacle CA (2008) Postnatal catch-up growth after fetal protein restriction programs proliferation of rat preadipocytes. Obesity (Silver Spring) 16: 2760–2763. 18833213 10.1038/oby.2008.417

[48] Ozanne SE, Nave BT, Wang CL, Shepherd PR, Prins J, et al (1997) Poor fetal nutrition causes long-term changes in expression of insulin signaling components in adipocytes. The American journal of physiology 273: E46–51. 9252478 10.1152/ajpendo.1997.273.1.E46

[49] Desai M, Guang H, Ferelli M, Kallichanda N, Lane RH (2008) Programmed upregulation of adipogenic transcription factors in intrauterine growth-restricted offspring. Reproductive sciences 15: 785–796. 19017816 10.1177/1933719108318597 PMC3444244

[50] Briana DD, Malamitsi-Puchner A (2009) Intrauterine growth restriction and adult disease: the role of adipocytokines. European journal of endocrinology/European Federation of Endocrine Societies 160: 337–347. 10.1530/EJE-08-0621 19095781

[51] Juge-Aubry CE, Somm E, Giusti V, Pernin A, Chicheportiche R, et al (2003) Adipose tissue is a major source of interleukin-1 receptor antagonist: upregulation in obesity and inflammation. Diabetes 52: 1104–1110. 12716739 10.2337/diabetes.52.5.1104

[52] Somm E, Henrichot E, Pernin A, Juge-Aubry CE, Muzzin P, et al (2005) Decreased fat mass in interleukin-1 receptor antagonist-deficient mice: impact on adipogenesis, food intake, and energy expenditure. Diabetes 54: 3503–3509. 16306368 10.2337/diabetes.54.12.3503

[53] Somm E, Cettour-Rose P, Asensio C, Charollais A, Klein M, et al (2006) Interleukin-1 receptor antagonist is upregulated during diet-induced obesity and regulates insulin sensitivity in rodents. Diabetologia 49: 387–393. 16385385 10.1007/s00125-005-0046-x

[54] Delahaye F, Lukaszewski MA, Wattez JS, Cisse O, Dutriez-Casteloot I, et al (2010) Maternal perinatal undernutrition programs a “brown-like” phenotype of gonadal white fat in male rat at weaning. American journal of physiology Regulatory, integrative and comparative physiology 299: R101–110. 10.1152/ajpregu.00604.2009 20463183

